# Context-Aware Naming and Forwarding in NDN-Based VANETs

**DOI:** 10.3390/s21144629

**Published:** 2021-07-06

**Authors:** Waseeq Ul Islam Zafar, Muhammad Atif Ur Rehman, Farhana Jabeen, Byung-Seo Kim, Zobia Rehman

**Affiliations:** 1 Department of Computer Science, COMSATS University, Islamabad 45550, Pakistan; zobia.rehman@comsats.edu.pk; 2 Department of Electronics and Computer Engineering, Hongik University, Sejong 30016, Korea; atif_r@outlook.com; 3 Department of Software and Communication Engineering, Hongik University, Sejong 30016, Korea; jsnbs@hongik.ac.kr

**Keywords:** NDN, context-aware naming, VANETs, NDN naming, forwarding, critical content dissemination, NDN-based VANETs

## Abstract

Vehicular ad-hoc network (VANET) is a technology that allows ubiquitous mobility to mobile users. Inter-vehicle communication is an integral component of intelligent transportation systems that enables a wide variety of applications where vehicles interact and cooperate with each other, from safety applications to non-safety applications. VANETs applications have different needs (e.g., latency, reliability, delivery priorities, etc.) in terms of delivery effectiveness. In the last decade, named data networking (NDN) gained the attention of the research community for effective content retrieval and dissemination in mobile environments such as VANETs. In NDN, the content’s name has a vital role in storing and retrieving the content effectively and efficiently. In NDN-based VANETs, adaptive content dissemination solutions must be introduced that can make decisions related to forwarding, cache management, etc., based on context information represented by a content name. In this context, our main contributions are two-fold: (i) we present the hierarchical context-aware content-naming (CACN) scheme for NDN-based VANETs that enables naming the safety and non-safety applications, and (ii) we present a decentralized context-aware notification (DCN) protocol that broadcasts event notification information for awareness within the application-based geographical area. Simulation results show that the proposed DCN protocol succeeds in achieving reduced transmissions, bandwidth, and energy compared to existing critical contents dissemination protocols.

## 1. Introduction

Advances in information and communication technology (ICT) in areas such as hardware, software, and communications enable its integration with the transport infrastructure to intelligently provide support for planning, designing, maintenance, and transport management. Vehicular ad-hoc networks (VANETs) allow ubiquitous mobility to mobile users. Inter-vehicle communication is an integral component of intelligent transportation systems (ITSs) [1,2]. The capability of in-vehicle technology enables a wide variety of applications where the vehicles interact and cooperate with each other, from safety and warning applications [3] to non-safety applications whose aim is to increase drivers’ convenience and efficiency by delivering services. Therefore, autonomous vehicles have been the subject of intense research recently [4]. Tesla, Google, Amazon, Uber, and automakers such as Ford, Volkswagen, and Audi are investing heavily in the research and development of autonomous vehicles that can provide services without driver attention. According to a report, as many as 8 million autonomous cars will be shipped in 2025 [5]. The biggest challenge posed by autonomous vehicles is how much data these vehicles will generate and communicate [6].

Contents associated with VANET’s applications when generated are associated with the time and location where generated. VANETs applications have different quality of service (QoS) expectations (e.g., latency, reliability, and availability) and transportation requirements in terms of delivery effectiveness [7,8,9]. Existing VANETs applications can be divided into two subgroups: non-safety and safety. A non-safety subgroup that is delay-tolerant includes applications whose primary goal is the delivery of services to clients and automates vehicle-related tasks. Applications under the safety subgroup include time-sensitive applications where vehicles exchange safety messages with each other or with roadside units (RSUs) to ensure safety. If the relevant information is not delivered in time, its usefulness is lost. For example, in the event of an automobile accident (post-crash notification application), information needs to be received at the nearby entities (vehicles and RSU) in a timely manner so that it can be transferred to the emergency center of the health maintenance organization. Otherwise, inefficient safety-related content dissemination could lead to life loss and disabilities. Moreover, such information is also necessary for nearby vehicles to make timely decisions on speed, lane change, etc. In case of a road hazard, information must be received at the nearby traffic police station within a short time. Otherwise, it could lead to traffic congestion, which could result in casualties, damage, and time wastage for the motorist and passengers. The time validity of non-safety applications (fee information relating to charging stations for electric vehicles and promotional messages in a road area) may extend for several hours. Different delivery priorities may be required by the traffic of the same type, e.g., video. For example, video delivery to a vehicle required for safe driving (to raise awareness related to the area of interest under bad weather) should take precedence over the delivery of a recent cartoon for a child. The latter video is less critical, but both videos demand quality of experience (QoE).

Ineffective data dissemination may trigger poor use of resources, increased delay in content delivery, and unavailability of content/services, resulting in degradation of user quality of expectation. Taking into consideration the VANETs characteristics, environment (urban, highway, and rural), and applications, efficient and effective content dissemination in NDN-based VANETs is a known problem and faces unique challenges [9,10,11].

Like mobile ad-hoc networks (MANETs), in IP-based VANETs, it is necessary to identify each vehicle with a unique address. The IP of vehicles changes as they move to new physical places. This creates interruptions in communication between vehicles [12]. The IP communication model is based on host-to-host communications, where one host requests it, and others provide a resource. Furthermore, IP-based routers do not support caching. In an infrastructure-less environment, autonomous vehicles will be unable to survive if traditional IP-based protocols are used. Recently, the concept of information-centric networking (ICN) has been proposed for future Internet architecture [13,14]. The main concept of ICN is to support content-oriented communication, not host-oriented. The content identifier does not depend on location. It allows in-network caching to reduce delays in retrieving content. Consequently, producer availability for retrieving data is not required. Many ICN-associated projects have been proposed, such as NDN/CCN [13,14]. To build a content-based connection model, all these architectures replace hosts with contents. Data-oriented network architecture (DONA) and network of information (NetInf) architectures are considered for fixed networks. NDN uses location-independent, variable-length, application-generated content names for retrieving and/or disseminating the content efficiently. Name-based communication does not require any central entity for name resolution. Therefore, provide better forwarding, especially in VANETs environments. Furthermore, in-network caching is particularly beneficial under intermittent connectivity and dynamic topology as it can facilitate content retrieval. Thus, by using the above benefits, NDN is more feasible for effective content retrieval in highly mobile and dynamic environments such as VANETs.

In NDN-based VANETs, the content’s name has a vital role in disseminating, storing, and retrieving the content effectively and efficiently [13,14,15]. Different VANETs applications have multiple, possibly conflicting, and different QoS expectations. Without a proper context-aware naming scheme, it is not possible to provide a scalable and effective content dissemination solution. The context information can be aggregated along with the information components such as type of content (such as safety, non-safety), scope, application type (e.g., post-crash notification), content format (text, audio, video), the location where content is generated, the time when content is generated, and network environment (rural, urban, and highway). While there has been work on naming for NDN-based VANETs, it tends to be narrow in scope and aims [15,16,17,18,19,20,21,22,23,24,25,26,27,28]. The existing NDN-based naming schemes cannot cater to a wide range of VANETs applications. Moreover, the current state of the art does not address the content fragmentation requirement. Most of the existing schemes do consider some of the context components such as time, location, application type [15,20,21]. However, naming in NDN is still subject to more investigation to be tailored for VANETs.

In NDN-based VANETs, adaptive content dissemination solutions must be introduced that can make dynamic decisions (related to forwarding, cache management, etc.) based on content context, spatial validity, time validity information [7], rather than treating each content in the same way. The most appropriate forwarding strategy can be chosen based on context and associated transportation requirements [29,30,31,32].

Different events on the road (e.g., post-crash notification, road work, etc.) can significantly affect traffic conditions. Dissemination of event notification to vehicles heading to the event site may help to make timely decisions such as to reroute or reduce speed. The conventional NDN takes advantage of the pull-based model, where data are being retrieved upon consumer request only. In most safety applications, the content is created based on event detection or in case of an emergency [9,10,11]. Furthermore, it is necessary to notify the vehicles prior to reaching the hazardous site. To respond to the minimum latency requirement, the current state of the art takes into account the push-based content model for safety-related content forwarding [23,33,34,35,36,37,38,39]. Pushing content is useful for disseminating content over the geographic area. However, the push-based content dissemination approach meets the safety application’s requirements (such as low latency, availability, reliability) with increased communication and storage costs. If the push-based model is considered for disseminating safety content, then in native NDN, the vehicle discards the unsolicited content upon reception. There exists work that uses the beacon messages to announce events or safety content [37,38]. Upon the reception of beacons, the vehicles create synthetic entries in the pending information table (PIT), which helps to accept the data pushed into the network. These schemes [23,33,34,35,36,37,38,39] overlook the suppression of duplicate messages while forwarding and exchanging the messages with other vehicles. In contrast to the works of [33,34,35,36,37,38,39], just the work of [23] discusses the content-naming scheme considered in their safety content forwarding scheme. The proposed naming scheme tends to be narrow in scope and can be used to represent just a particular application scenario [23].

In most safety applications, notification messages containing only the context-aware names will be sufficient for vehicles to make the decision timely. For example, in safety applications (such as dangerous road warning, work zone warning, emergency vehicle warning, post-crash notification), all vehicles moving to the hazardous site need a contextual alert to make timely decisions such as to reroute. Even such an alert will be adequate for the emergency vehicles to reach the hazardous site. In such applications, the notification messages with the context-aware content name will reduce the need to attach content to the messages. Research efforts should be focused on the development of context-aware content-naming (CACN) solutions for NDN-based VANETs that must allow representing different applications, not just a particular application scenario. The CACN scheme will help to introduce adaptive content dissemination solutions that will allow selecting solution based on content context information rather than treating each content in the same way.

### Contributions

More specifically, the major contributions of this paper can be summarized as follows:

- The hierarchical context-aware content-naming (CACN) scheme for NDN-based VANETs is proposed. CACN scheme allows naming the safety and non-safety applications contents. It enables the entities to retrieve context and content-specific information from the content name, which allows effective and efficient context-aware decisions related to content forwarding and caching;
- The CACN scheme exploits the coding scheme to represent most of the content name components, which allows addressing communication and storage complexity;
- In this work, we focus on the dissemination of safety-related information with reduced latency. To this end, we present a decentralized context-aware notification (DCN) protocol that broadcasts event notifications for information awareness within the application-based geographical area. The dissemination of messages in the geographic area is controlled by exploiting the spatial and time validity requirements of the application. The viability of a DCN protocol for safety (critical) content notification is enhanced by presenting the broadcast control mechanism that assigns priority to the vehicle based on its location, angular position, and distance to the event site;
- Simulations in an NS-3-based NDN simulator (ndnSIM) [40,41] to check the performance of the proposed scheme with relevant and state-of-the-art schemes [23,39].

The remaining document is organized as follows: Section 2 describes the applications of VANETs; Section 3 discusses the relevant work; Section 4 discusses the context-aware naming scheme for NDN-based VANETs; Section 5 present a context-aware forwarding scheme for safety contents; Section 6 presents the performance analysis; and Section 7 concludes our work.

## 2. VANETs Applications

Typical VANETs applications can be broadly classified into two categories: (i) non-safety and (ii) safety applications.

### 2.1. Non-Safety Applications

Comfort and entertainment applications are called non-safety applications that aim to improve drivers’ and passengers’ comfort levels and make travel more pleasant. These applications require high bandwidth. Moreover, such applications do not demand high availability as information is not needed by all the vehicles but on-demand according to user choice [3,13,14]. Normally, the typical requirements of these applications are reliability, availability, and connectivity. These applications and services are not bound to the limited geographical area and low latency requirements. Therefore, such applications are also termed as non-critical applications. Such applications are delay-tolerant because they have no stringent real-time requirements. However, reducing the delay and packet loss for non-safety applications would improve service quality. Spatial validity represents the extent of a geographical area in which the information is required, valid, and valuable (termed as spatial validity of the content) [9]. Spatial and time validity information can be used by vehicles to determine, for example, what to cache, and whether to participate in disseminating messages. The spatial and time validity requirements of an application might change depending on the road type where vehicles are driven [3,8,10]. The reason is that a vehicle must comply with the road network’s mobility pattern. Some of the non-safety applications are as follows:

○ *Multimedia file sharing*: Such applications allow users to share files such as music and movies. The spatial validity for such an application is 1 km, and the temporal validity is 10 min. The service consumers are required to subscribe to such a service [13,14].
○ *Commercial advertisement*: Service area announcements, restaurants, and other businesses can use an RSU to send promotional messages to vehicle drivers in their communication range. The spatial validity for such an application is 1–5 km, and the temporal validity is 1–10 days [13,14]. The user may only want to receive the selected brand’s advertisement not all, so it varies from vehicle to vehicle.
○ *TrafficNavigationmap*: This application is only required by subscribers. The spatial validity is 10 km and time validity is 30 min [13,14].

### 2.2. Safety Applications

The quality of service (QoS) required by the safety applications is close to real-time. The delay in information dissemination could lead to life loss and disabilities. Such information is also necessary for nearby vehicles to make timely decisions on speed, lane change, etc. Therefore, such applications are also termed as critical applications. Another requirement is reliability. All vehicles close to the hazard need to be alerted. Vehicles use the supplied information to make decisions during the trip. For example, all vehicles in the hazardous neighborhood are interested in receiving safety messages, compared with non-safety applications where a small number of vehicles are interested in commercial advertisements. Car collisions are currently one of the most common causes of death. Road safety applications primarily provide drivers with assistance in avoiding vehicle collisions and reducing crash death ratios [3,13]. The focus of such applications is to assist drivers by providing time-sensitive traffic information that enables drivers to avoid accidents from occurring in the first place.

In a post-crash notification application, a vehicle involved in a road accident broadcasts warning messages to request support from highway patrol as well as to inform the impending vehicles so that they can make timely decisions about changing the lane or route. These applications have lesser bandwidth requirements but have localized spatial scope regarding the extent of a geographical area in which the information is required, valid, and valuable (termed as spatial validity of the content) [14]. Localized spatial validity indicates that the spatial scope of such messages is limited. For example, the speed-warning message is only valid to vehicles approaching the sharp road turn, say within 100 m. The time validity for 30 s means that the message should be considered valuable if its time freshness is less than time validity [7,9,14]. The time freshness represents the time in transit after message creation. The receiving vehicle calculates time freshness based on the current time and time of creation specified in the message. In a post-crash notification application, if the received message time freshness is less than the specified time validity (say 30 s), then it can be considered valuable, and necessary actions should be taken, such as participation in message dissemination. Existing work suggested 30 s as time validity for post-crash notification application, but it is simply a rough specification and can be changed based on requirements. Some of the safety applications are as follows:

○ *Post-crash notification:* A vehicle involved in an accident or spectator vehicle sends warning messages in a broadcast to approaching vehicles (its time validity is 30 s within 500 m) [13,14].
○ *Work zone*: This information is needed by all the vehicles in the range of 0–1 km moving toward the work zone so that they can choose alternate routes. The time validity is construction duration [13,14].
○ *Dangerous road warning:* This information is needed by all the vehicles in the range of 100 m moving toward the work zone so that they can choose alternate routes. The time validity is 10 s [13,14].
○ *Emergency Vehicle Warning*: This information is needed by all the vehicles in the range of 500 m. The time validity is 10 m [13,14].
○ *Highway information:* This information is needed by all the vehicles in the range of 5 km. The time validity is all day [13,14].
○ *Road congestion information:* Such a warning message by the vehicle facing road congestion helps the other vehicles to make timely decisions such as to change lanes, etc. Only the vehicles within the 5 km range of the hazard need this information. Information is valid for only for 30 min [13,14].

## 3. Related Work

This section reviews the work on naming schemes and safety content dissemination in NDN-based VANETs.

### 3.1. Naming Schemes for NDN-Based VANETs

This section briefly discusses the proposed naming schemes for vehicular NDN. Table 1 compares existing literature on naming schemes for NDN-based VANETs.

Wang et al. [15] proposed a naming scheme for disseminating traffic information. The format used for representing the name is as follows: /traffic/geo-location/timestamp/datatype/nonce. The traffic component represents the traffic application ID. Moreover, geo-location is represented with the format road ID/direction/section number. Datatype component represents the type of data, e.g., speed, closed lane, etc. A nonce is included to distinguish between publishers. The authors discuss that time and location information are relevant in most contexts. Pesavento et al. [16] proposed a hierarchical naming scheme that maps bi-dimensional geographic areas into a uni-dimensional naming scheme using an encoding algorithm. Each coordinate of the location (x; y) consists of digital bits. The pairing function is used to convert the location into the one-dimensional sequence $c_{i}$, each used as a separate name component (/NDN/bit/parking/……. /$c_{1}/c_{2}/../c_{n}$). Yan et al. [17] proposed a hierarchical naming scheme for NDN-based VANETs. The name structure is divided into three sections: destination location, source location, and next location along the direction from the source location to the destination location. Furthermore, this naming scheme allows the aggregating name of the content from the same city, district, or street.

Amadeo et al. [18,19] presented forwarding schemes that forward based on the content priority. The vehicular data traffic is divided into two priority categories, i.e., high and low. Priority tag is used at the start of the naming prefix. For example, to request urgent road traffic information for a vehicle, the content name is represented as follows: /high/trafficinfo/streetxy/km20/. Two information components are considered by the naming module to assign priority categories to the application: (i) application latency requirements and (ii) user-defined preferences related to cost and bandwidth. High-priority content is forwarded using 3G/LTE and IEEE 802.11 OCB. Low priority content is forwarded using IEEE 802.11 OCB. The scheme does not perform well in a highly dynamic environment due to frequent rebroadcast because of not considering a proper FIB updating mechanism for non-requested contents.

Liu et al. [20] proposed a hierarchical naming scheme, where the content name is represented using the format: /application/geo-reference/temporal-field/nonce. The application component represents the application to which the content is associated. The geo-reference component indicates the location. The temporal field represents the content time when published. The nonce is used to identify different data providers. This work also presented a forwarding solution using consumer location and road trajectory information for forwarding decisions. The scheme used geotags for interest and data messages. The requestor, before forwarding the interest message, adds its location so that the content can be retrieved at the new expected location of the requester. 

Chowdhury et al. [21] proposed a scheme to identify false information dissemination malicious vehicles. The naming is represented using the format: /application-prefix/datatype/data-location/name-marker/vehicle-name/timestamp. The application prefix component represents the application kind, e.g., real-time traffic or an accident prevention application. Datatype component used to specify the data type, e.g., the status of the road or the arrival of emergency vehicles. The authors specified that the location component can have multiple subcomponents such as country/state/city/road/road section. The name-marker component is used as a tag to represent that the next component is the vehicle name. Moreover, the timestamp component represents the time to which the content is associated. The time can be represented using a range or single value. In contrast to [18], this work includes the component vehicle name that produced the data to bind the data with its original vehicle producer.

Modesto et al. [22] proposed a hierarchical naming scheme. Content name is represented using the format: class/class-specific category/application/primary identifier/contextual identifier/additional tags. Applications are divided into three categories: (i) safety, (ii) transit, and (iii) infotainment. The global context is represented using class-specific category and application components. The application component identifies the type of services. Finally, primary identifiers and contextual identifiers are exploited for representing application context, such as speed/location. Furthermore, the additional tags component is used to represent information such as radius range, content resolution information. Ullah et al. [23] presented a hierarchical naming scheme for naming safety contents. The content name includes the producer vehicle ID, roadside unit (RSU) ID, vehicle speed, and direction (such as /VID/RSUID/Speed/direction/payload). A push-based model is considered for disseminating safety content. In the work of [23], the vehicles in the range of RSU having ID specified in the content name only participate in its dissemination. The vehicles push the data in the area covering the radio range of RSU.

Quan et al. [24] designed a hybrid naming scheme for naming multimedia contents. The content name is comprised of three components: hierarchical routable prefix (HRP), flat content identifier (FCI), and primary attribute labels (PALs). The HRP represents information required for forwarding and routing, such as location. FCI uniquely identifies the content generated using hash, used for efficient cache retrieval. Finally, PALs represent contextual information about the content, such as time, priority level, and caching strategy. Moreover, this work proposes an effective cooperative caching algorithm for multimedia content to provide better user QoS in highway scenarios. The author proposed two schemes, partner-based and courier-based, given the lane and speed of the vehicle. Bouk et al. [25,26] proposed a hybrid naming scheme for vehicular ICN. The name is comprised of three components: (ii) owner/producer information, (ii) content information, and (iii) hash value. The first two components are hierarchical. The content information represents information about the content format, location, and time. Content is uniquely identified by the hash component.

Prates et al. [27] proposed geographic-based naming and the zone-based forwarding scheme. Location-based content naming is used for efficient retrieval of content. Interest forwarding is based on the relative location of the requested content. The following structure is used for representing name: /longitude/latitude/application/timestamp. An application specifies the content type, such as movies and traffic status. The vehicles in the dissemination zone are allowed to store and forward the received data messages. A vehicle will be selected as a forwarder if it belongs to the zone, which the requested data belong to.

Yu et al. [28] proposed a hierarchical naming scheme for VANETs. Name is represented using the following format: /category/service/additional info. The data services are classified into three categories: popular public, popular private, and unpopular. Service is used to differentiate between providers, and additional info is used for content identification. This work assumes that the urban map is hierarchically divided into partitions. Each vehicle, based on its location coordinates, decides to which partition it belongs. Leaf partitions correspond to the road segment. Bloom filters (BFs) are used to announce the content name. 

Most existing naming schemes do not consider coding schemes that can help reduce content name size and support the inclusion of meaningful content contextual information. Some state-of-the-art naming schemes are targeting only specific scenarios [16,17,23,28]. The naming scheme presented in the work of [18,19] allows specifying QoS and latency requirements by adding a priority tag to the content name. In the work of [21], the authors do not specify the content-related information that can help identify the QoS and latency requirements, while in the work of [18,19,23,27], the focus is on geographic-based forwarding. Moreover, there does not exist any work that addresses the content fragmentation requirement in the content name. 

### 3.2. Safety Content Dissemination in VANETs

To address the lower latency requirement in disseminating safety-related content, the existing state of the art exploits one of the following approaches: (i) push-based forwarding [23,34,35,36,39], (ii) beacon message with push-based data forwarding [37,38], and (iii) event message with push-based forwarding [33]. Pushing content is useful for disseminating content over the geographic area. In the work of [23], whenever a vehicle receives a data packet, it will participate in its dissemination if it is lying in the RSU radio range whose ID is mentioned in the content name. In the work of [33], an event message is generated to advertise the existence of safety content within the network.

Proactive caching aims to predict the next location of a mobile device based on the mobility pattern [34,35] to satisfy the Interest during the handover. To address the requirement of seamless support of mobility in NDN, in [34] scheme is presented, which exploits location and data traffic prediction. Abani et al. [35] proposed an entropy-based proactive strategy to predict a mobile entity’s next location. Markov predictor is used to locate a mobile vehicle’s next location. In the work of [34,35,36], the content is proactively pushed in the network for caching ahead of handover. Lehmann et al. in [36] proposed two push-based caching schemes, where data provider pushes predicted contents to the network when handover is imminent. The first scheme pushes the content to the other nodes in the network at every movement, not considering whether nodes are interested or not. In the second scheme, the producer sends suggestion to the nearby network nodes to cache the content. There exists work that uses the beacon messages to announce events or safety content [37,38]. Upon the reception of beacons, the vehicles create synthetic entries in the pending information table (PIT), which help to accept the data pushed into the network. Flooding of beacon messages in NDN-based VANETs can quickly increase PIT size. The problem becomes worse in the case of beacon packet long lifetimes, which increase the number of stale entries in PIT. Such PIT entries will not only waste PIT storage but also increase lookup time for PIT entries. These events are very common in VANETs due to high mobility. In the work of [38], the vehicles participate in the dissemination of beacon and data messages till the destination is not reached, which is the RSU location. 

Niari et al. [39] presented extended content-centric network (ECCN) architecture to improve data access by reducing the latency for the critical contents. Context is represented in terms of the content type. Two content stores are introduced at the same node, named local content store (LCS) and neighbor content store (NCS). NCS stores all the safety contents received from the neighboring vehicles and produced by itself. In contrast, the LCS is exploited by the vehicle to save non-safety contents. Like the work of [23], a push-based forwarding scheme is considered for the dissemination of safety content. Flooding of the content is controlled using time to live (TTL). The scheme overlooks the suppression of the duplicate messages while forwarding and exchanging the content store with other vehicles. The vehicle is responsible for sharing the safety content with neighbors upon reception. 

In the work of [23,34,35,36,39], the authors did not discuss how to address the unsolicited content problem. To control flooding, in the work of [33,39], the number of hops is specified in the data messages. In the work of [33,39], when a vehicle receives a data packet, it will participate in its dissemination only if the TTL in the packet is greater than zero. Each time upon receiving the same (duplicate) message from the neighbors, a vehicle rebroadcasts it after decrementing the TTL. No suppression scheme is proposed to address broadcast storm problem [23,33,34,35,36,37,38,39].

Among the existing work [23,33,34,35,36,37,38,39], which presented push-based forwarding schemes for disseminating critical content, only the work of [23] discussed the naming scheme. The naming scheme targeted only specific application scenarios. The content name includes the producer vehicle ID, roadside unit (RSU) ID, vehicle speed, and direction. The vehicles in the range of RSU having ID specified in content name push the data in the area covering the radio range of RSU. Each time upon receiving the same (duplicate) message from the neighbors, a vehicle reforwards it as long as it satisfies the validity check (lying in the RSU range whose ID is mentioned in the content name).

The push-based content dissemination approaches [23,33,34,35,36,37,38,39] meet the requirements of the safety application (such as low latency, availability, and reliability), but with increased communication, computing, and storage requirements. Our work also proposes adopting the push-based model for safety information dissemination. However, the focus is to send the notification message with a context-aware content name. In this work, we present context-aware content-naming (CACN) scheme for VANETs. Our work focuses on the vision that in safety applications, context-aware content names in notification messages will be sufficient for the participating entities to take necessary actions. Context-aware content name reduces the need to attach content as in most of the safety applications; the vehicles need a contextual alert. Moreover, our CACN scheme allows the introduction of adaptive content dissemination solutions in NDN-based VANETs. We also present a decentralized context-aware notification (DCN) scheme that pushes event notification for information awareness within the application-based geographical area. The viability of a DCN scheme for safety content notification is enhanced by presenting the broadcast control mechanism. 

## 4. Proposed Context-Aware Content-Naming (CACN) Scheme

In this section, we will present the context-aware content-naming (CACN) scheme. CACN scheme subdivides the name hierarchically into components. The initial component in the CACN scheme is content type (*CT*), enabling the efficient implementation of content prioritization schemes considering application type. This segregation of the content type is based on the communication requirement of the data, such as reliability, latency, and availability. Like native NDN, the content name components are separated with ‘/’. The subcomponents are segregated by the separator ‘:’ rather than ‘/’ for easier identification of names major and subcomponents. CACN scheme broadly divides the applications into two categories: safety and non-safety. These categories include VANETs applications having varying requirements, including reliability, latency, and availability.

**Figure 1:**
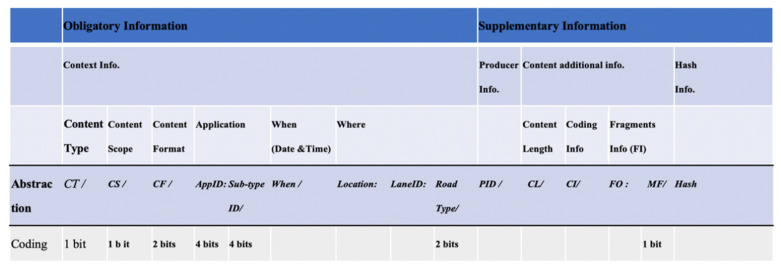
CACN naming format.

Figure 1 presents the CACN scheme. The content name is divided into two partitions: (i) obligatory and (ii) supplementary. The obligatory part holds the contextual information about the content. The CACN scheme allows the entities to derive received content properties from its name. The context information is aggregated along with the following components: content type (CT), content scope (CS), content format (CF), application, when, and where. CT represents the kind of content, i.e., safety or non-safety. Our naming scheme supports handling the content lookup more efficiently and effectively by allowing segregating the content according to CT. CS represents the scope of the content, i.e., local or global. The CF field represents content format, which is divided into four categories: text, audio, image, and video. The application component has two subcomponents: Application ID (AppID) and *Sub-type ID*. AppID uniquely identifies the VANETs application. *Sub-type* defines the nature of application such as news, sports, cartoons, and movies. The content name’s supplementary partition holds the information about the producer and vital information about the content such as fragments information (FI), its coding information, and content size, etc. Producer identity (PID) holds vehicle identity (original or anonymous) information that generated the content, required to address the security requirements [21,42,43]. Due to the large and distributed nature of VANETs, mostly the communication occurs between vehicles that are strangers to each other. Moreover, there may exist malicious vehicles in the network that may broadcast false messages due to different reasons, including business gains, personality/habit, victim exploitation, irresponsibleness, and randomness. Calculating the trustworthiness of both data and vehicles will help vehicles to make correct, timely decisions to avoid dire consequences of acting on false messages from malicious vehicles. Therefore, we included PID in our content name.

The CACN scheme exploits the coding scheme to represent most of the context information components, which include: (i) CT, (ii) CS, (iii) CF, (iv) AppID, (v) *Sub-Type ID*, (vi) RoadType, and (vii) more fragments (MF). The first byte of the content name holds information about the following name components: (i) CT, (ii) CS, (iii) CF, (iv) AppID. From the first byte of a content name, effective and efficient context-aware decisions related to the content forwarding, caching, and lookup can be made. The coding information helps support efficient lookup with minimum latency. The existing current state of the art [7,8,9] suggested 10 s as time validity for dangerous road warning applications. The current state of the art suggests similar values for spatial and time validity for each network environment (urban, rural, and highway). Our CACN enables the representation of the network environment as part of the name. The road type component is added in the CACN to be used, if necessary, to specify the spatial validity and time validity for applications based on road type. Our CACN allows us to introduce adaptive content dissemination solutions in NDN-based VANETs, that can make dynamic decisions (related to forwarding, cache management, etc.) based on content context (specified in the content name) information, rather than treating each content in the same way.

### 4.1. CACN Attributes and Coding

This section describes the CACN components and the coding scheme used for their representation.

#### 4.1.1. CACN Obligatory Part

As depicted in Figure 1, the compulsory part of the CACN is divided into the following components:

- *Content Type (*CT*):* CT segregates the content into two basic types: safety and non-safety. For this segregation, only the first bit will be used. The safety content is represented by 0, while the non-safety content is represented by 1;
- *Content Scope (*CS*):* The second bit will be used to represent CS. The CS can be local, represented by 0, or global, represented by 1. VANETs applications can also be distinguished by their scope, i.e., whether they provide communication across a large geographical area or are local only. The applications having local scope represent a restricted spatial scope and a limited temporal scope. Examples include post-crash warnings and multimedia sharing. The global scope represents that the content can be disseminated within the larger geographic area. Examples include distributed games.
- *Content Format* (CF): The CACN scheme supports four content formats: text, audio, image, and video. CF help manages the content according to its quality of service (QoS) requirements. With 2 bits, four states can be supported: 00, 01, 10, or 11. The 2-bit state 00 is used to represent text notification or information, 11 for video content, 01 for pictorial information, and 10 for audio-type content;
- *Application:*
  - *Application ID (*AppID*):* Four bits are used for application specification. CACN naming scheme allows representing 32 different applications uniquely. For this purpose, *CT* and AppID content-naming components are considered in combination. A total of 4 bits for AppID, allow representing 16 safety and 16 non-safety applications where each application can have 16 subtypes. The general segregation of applications is safety and non-safety categories, using the bit coding scheme is as follows:
    - When *CT* = 0
      ○ AppID = 0000 used to represent post-crash notification
      ○ AppID = 0001 used to represent work zone
      ○ AppID = 0010 used to represent dangerous road warning
      ○ AppID = 0011 used to represent emergency vehicle warning
      ○ AppID = 0100 used to represent highway information
      ○ AppID = 0101 used to represent road congestion information
      ○ …………………………………..
    - When *CT* = 1
      ○ AppID = 0000 used to represent multimedia file sharing
      ○ AppID = 0001 used to represent commercial advertisement
      ○ AppID = 0010 used to represent traffic navigation map
      ○ …………………………………….
  - *Sub-Type ID (Sub-Type ID):* Four bits are used for application sub-type specification. The general segregation of application sub-type, using the bit coding scheme is as follows:
    - When *CT* = 0, AppID=0000
      ○ *Sub-Type ID* = 0000 used to represent head-on collisions;
      ○ *Sub-Type ID* = 0001 used to represent hit from behind;
      ○ *Sub-Type ID* = 0010 used to represent hitting the driver in front;
      ○ *Sub-Type ID* = 0011 used to represent hide collisions;
      ○ …………………………………………..
    - When *CT* = 1, AppID=0000
      ○ *Sub-Type ID* = 0000 used to represent scenic landscape;
      ○ *Sub-Type ID* = 0001 used to represent drama;
      ○ *Sub-Type ID* = 0010 used to represent cartoon;
      ○ *Sub-Type ID* = 0011 used to represent music;
      ○ …………………………………………..
- When*:* When represents the content creation time used to hold the content time of creation. We have not designed a coding scheme for this naming component. For the sake of flexibility, we have left it open-ended. However, it can be represented using Unix base timestamp format (length 4–8 bytes [44]);
- *Where: Where* is comprised of Location, LaneID, and RoadType. Location represents the location where the content is generated or to which it belongs. CACN scheme is based on global positioning system (GPS) location, and the vehicles obtain their location information via GPS. For example, 31.418474/73.079147 information represents the clock tower Faisalabad, Pakistan location. The location can be represented using 8 to 16 bytes [45];
- RoadType is used to represent the network environment. LaneID represents the lane number. Road type (urban, rural, or highway) represents VANET’s conditions such as density and network traffic. Two bits are used for RoadType specification: state 00 to represent a rural road, 01 for urban road, 10 for highway, and 11 for the street. LaneID can be calculated using one of the schemes presented in recent work on LaneID computing [46].

#### 4.1.2. CACN Optional Information Part

- *Producer ID (PID):* The PID can hold information such as registration number, MAC address, anonymous identity (pseudonym). We have not designed a coding scheme for this naming component, but it can be represented using 6 to 18 bytes [23];
- *Content Length (CL*): CL holds information about the content size. We have not designed a coding scheme for this naming component. It can be represented using 2 to 4 bytes as represented in IP networks [47];
- *Coding Info (CI):* Holds information about the content format, such as resolution encoding and sampling information. We have not designed a coding scheme for this naming component but can be represented using the scheme exploited in [48];
- *Fragments Information (FI):* In a case where the data cannot be forwarded using a single packet because of its size, then data are divided into fragments. The fragments may be received out of order; therefore, the fragment offset (FO) information component is exploited while reassembling the received fragments. The FO contains fragment offset information to identify the fragment’s starting position in relation to the original packet. FO is set to 0 in the first packet. For remaining fragments, FO is computed in units of 8-byte blocks as computed in IP networks [47]. This component will be empty in case the content is not divided into fragments. More fragments (MF) flag represents that there exist more fragments after the current fragment for a packet. MF value is either 0 or 1. The value 0 represents that it is the last fragment of the specific content;
- *Hash:* Hash-tag is added to uniquely identify the content, generated using the hashing algorithm, either MD5 or SHA-1. It can be represented using 12 to 16 bytes.

### 4.2. Use Case Scenarios in NDN-Based VANETs

#### 4.2.1. Non-Safety Content

**Figure 2:**
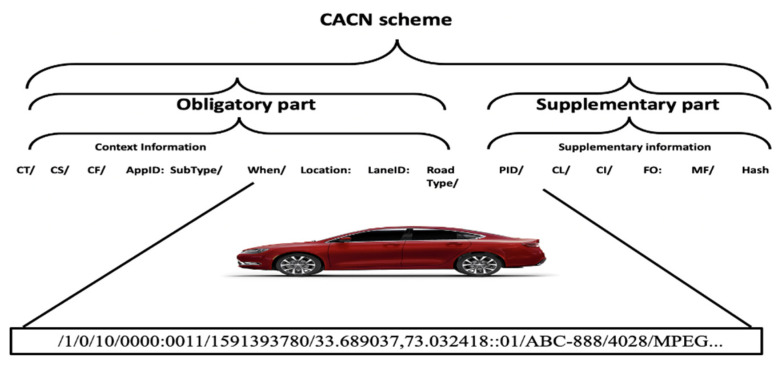
Example scenario where the non-critical content is generated by vehicle.

Figure 2 shows an example scenario where the vehicle produced non-safety music content while moving from an urban area (Markaz G-9, Islamabad, Pakistan). The vehicle gets its location information via GPS. The content is generated at location (1323201600;1323205200) on Friday, 5 June 2020, 9:49:40 PM GMT+05:00 (Unix timestamp= 1542193562000). The producer vehicle has registration number ABC-888. The content has a local scope, audio format, and multimedia file sharing application type. The content is not divided into fragments. The audio content size is 4 KB, and the format is MPEG. The generated content name prefix is as follows: /1/0/10/0000:0011/1591393780/ 33.689037,73.032418::01/ABC-888/4028/MPEG///....

#### 4.2.2. Safety Content

Let us consider an example scenario where the vehicle produced post-crash video content on the urban road at location (1323201600;1323205200) on Friday, 5 June 2020, 9:49:40 PM GMT+05:00 (Unix timestamp = 1542193562000). The producer vehicle has registration number LH-888 and is in lane 2. The content has a local scope, video format, and post-crash notification application type. The content is not divided into fragments. The content size is 1 MB, and the format is 3GP. The generated content name prefix will be as follows:

/0/0/11/0000:0000/1578808800/33.653006,73.157438:lane2:01/LH-888/1000000/3GP/….

#### 4.2.3. Content Fragmentation

Let us consider an example scenario where the vehicle produced a large-size non-safety cartoon content while moving from an urban area Markaz G-9, Islamabad, Pakistan (GPS location: 1323201600;1323205200) on Friday, 5 June 2020, 9:49:40 PM GMT+05:00 (Unix timestamp = 1542193562000). The producer vehicle is having a registration number LH-888. The content has a local scope, video format, and multimedia file sharing application type. The movie is divided into small fragments considering the maximum transmission unit (MTU) allowed on the outgoing face. Let us suppose the size of the video is 4000 bytes and the MTU is 2000 bytes. Let us suppose that the content name size and other attributes in the data message are 100 bytes. Three fragments will be required to transmit the video.

- The first fragment will contain 1900 bytes of the video content. The fragment offset will be zero, and MF will be set to 1. The context-aware content name for the first fragment will be:
  /1/0/11/0000:0010/1591393780/33.689037,73.032418::01/ABC-888/4000B//3GP/0:1/....
- The second fragment will contain 1900 bytes of the video content. The fragment offset will be (1900/8)=237.5, and MF will be set to 1. The context-aware content name for the second fragment will be:
  /1/0/11/0000:0010/1591393780/33.689037,73.032418::01/ABC-888/4000B//3GP/ 237.5:1/....
- The third fragment will contain 200 bytes of video content. The fragment offset will be (3800/8)=475, and MF will be set to 0. The context-aware content name for the third fragment will be:
  /1/0/11/0000:0010/1591393780/33.689037,73.032418::01/ABC-888/4000B//3GP/ 475:0/…..

CACN scheme handles the content lookup more efficiently by allowing seclusion according to content type (i.e., safety and non-safety content). Furthermore, it also allows prioritizing the content delivery based on CT, or/and CF, or/and AppID. The AppID, CF, CI components allow identification of the basic communication requirements of the requested content. In addition, the AppID, sub−typeID, CF components support prioritizing the content. Non-safety content communication requirements differ from safety content. Safety applications have fewer bandwidth requirements but have limited spatial scope concerning the extent of a geographical area in which the information is required, valid and valuable. Our scheme addresses non-safety contents as well by permitting to insert the necessary information such as coding information, size, and content fragmentation. Moreover, CT, AppID, RoadType helps to associate the spatial and temporal scope with the application. The road can be of type rural, urban, or highway. Existing work [7,8,9] just emphasizes associating single spatial scope and temporal scope with an application without considering the environment. However, our naming scheme supports linking different spatial scope and temporal scope with the same application considering road type/environment. For example, if the spatial scope of the post-crash notification application is 500 m in urban areas, then on highways, the spatial validity might be by 750 m due to high speed. Like existing work [7,8,9] in Section 5, we considered that an application is associated with a single spatial and temporal scope.

## 5. Context-Aware Forwarding Scheme for Safety Contents

In this section, we present the decentralized context-aware notification (DCN) protocol that broadcasts information about an event for awareness within the application-based geographical area. In this approach, whenever an event occurs, it notifies the nearby entities about the existence of the content. In the real-world scenario, when an accident occurs, then an event (e.g., the release of an airbag) triggers a system to send DCN messages to inform nearby entities [23]. In our scheme, the producer vehicle is immovable and sends DCN messages to the nearest RSU and vehicles. The DCN protocol aims to reach as many entities as possible quickly by generating less possible overhead in the network. The proposed DCN protocol performs better for safety applications, where the DCN message is intended for all the vehicles that are lying in the specific geographical area (as required by the specific application represented by spatial validity).

The DCN message contains the content name prefix and the location of the entity that forwards the DCN message (termed as the previous node). In the previous section, we proposed a context-aware hierarchical naming scheme for NDN-based VANETs that effectively reduces content name size with coding. The content name provides meaningful information about context to entities, thus optimizing network use. Like native NDN, each entity includes three data structures. The forwarding information base (FIB) data structure stores the forwarding information by maintaining a mapping of the content name prefixes and the interfaces (Face) through which the data packets can be forwarded. The pending interest table (PIT) stores the currently pending interests and their incoming interface that have not yet been served. The content store (CS) caches the content objects according to a caching policy.

In the work of [39], hop count is used to control the dissemination of the message, whereas, in the work of [23], the area-of-interest (RSU radio range) concept is exploited. The DCN protocol aims to reach as many entities as possible quickly by generating less possible overhead in the network. In our work, each vehicle can participate in the dissemination of the received DCN message only if it meets the spatial and time validity. To check the spatial and time validity of DCN messages, each vehicle is equipped with a spatial and temporal scope (STS) table. The STS table has five attributes: (i) *CT*, (ii) AppID, (iii) spatial scope (SS), (iv) temporal scope (TS), and (v) road type. As discussed in Section 2, each VANETs application is associated with spatial and time validity. The spatial and time validity for an application may vary based on the road type.

On reception of DCN message, the vehicles store in their forwarding information base (FIB) content name and the incoming interface. Every vehicle that receives a DCN message (DCN_msg) verifies whether a similar entry already exists for the name prefix specified in the received DCN_msg (Line 3 of Algorithm 1). If it is a duplicate message, the vehicle inhibits itself from forwarding the DCN message. If there is no such entry, then it first verifies the spatial and time validity of the received message.
**Algorithm 1**: DCN message dissemination protocol// STSTable   Spatial and Temporal Scope table // DCN message (DCN_msg) include content name (CACN), previous node location (PrevNodeLocation)   If (ChkForDuplicate(DCN_msg.CACN)  !=TRUE) then       // Searching application specific spatial and temporal scope in STS table       // DCN_msg.CACN.CT  content type specified in Context aware content name       // DCN_msg.CACN.AppID  AppID specified in Context aware content name       AppTempScope←TS_STSTable (DCN_msg.CACN.CT,   DCN_msg.CACN.AppID)       AppSpatialScope←SS_STSTable (DCN_msg.CACN. CT,   DCN_msg.CACN.AppID)       // Computing time freshness, and distance to hazard at current Vehicle       TimeFreshness←TimeInTransit (DCN_msg.CACN.When, CurrentDateTime());       CurrNode_DistHazard←Distance (DCN_msg.CACN.Where.Location, CurrentLocation());        If (TimeFreshness<  AppTempScope)         If (DistToHazard<  AppSpatialScope)          // *Computing previous entity (forwarder) distance to hazard*
        PrevNode_DistHazard←Distance (DCN_msg.CACN.Where.Location, DCN_msg.PrevNodeLocation);         Angle_PrevNode←AngleCompute(DCN_msg.PrevNodeLocation, CurrentLocation());           If (RedZone(Angle_PrevEntity)) $\;\;\;\;\;\;\;\;\;\;\;\;\;\;\;\;\;\;\;\;FrwdTime\leftarrow Delay_{Max}*\left(1-\frac{\;PrevNode\_DistHazard-\;CurrNode\_DistHazard}{RR_{max}}\right)+Delay_{random}\left(0,i\right)\;$           Else $\;\;\;\;\;\;\;\;\;\;\;\;\;\;\;\;\;\;\;\;FrwdTime\leftarrow Delay_{Max}*\left(1-\frac{\;PrevNode\_DistHazard-\;CurrNode\_DistHazard}{RR_{max}}\right)+Delay_{random}\left(i,j\right)\;$               SetDCNTimer(FrwdTime)   Else     Drop DCN_msg     StopDCNTimer(FrwdTime)


Let us suppose that the received DCN message is related to the application with AppID= $App_{i}$. Based on the CACN components (CT, and AppID), the STS table lookup is carried out to determine the spatial and temporal scope of the application (Equation (1), Equation (2)). The CACN component RT can also be used if spatial and temporal scopes for applications are stored in the STS table based on road type.

(1) AppTempScope←TS_STSTable(DCN_msg.CACN.CT, DCN_msg.CACN.AppID)          

(2) AppSpatialScope←SS_STSTable(DCN_msg.CACN. CT, DCN_msg.CACN.AppID)       

The vehicle computes time freshness based on current date and time and When component using Equation (3). The time freshness represents time in transit after DCN_msg creation, computed to verify time validity requirement.

(3) TimeFreshness←TimeInTransit (DCN_msg.CACN.When, CurrentDateTime())     

Time validity of the received DCN message is satisfied if the computed TimeFreshness is less than the AppTempScope of the $App_{i}$ given in the STS table (as shown in Algorithm 1). If the time validity is satisfied, a new FIB entry is added, and the receiving vehicle schedules to forward it using the deferred timer. Unix base timestamp format is exploited to store the date and time of creation in the Where name component in CACN.

(4) CurrNode_DistHazard←Distance (DCN_msg.CACN.Where.Location, CurrentLocation())

To verify spatial validity, the distance from the vehicle current location to the location where the content was produced (mentioned in When name component) is computed (Equation (4)). The spatial validity of the received DCN message is satisfied if the computed distance is less than the AppSpatialScope of the $App_{i}$ given in the STS table. Deferred timer concept and a duplicate suppression mechanism are being introduced to avoid the broadcast storm problem (unnecessary retransmissions). The forwarding strategy is based on the following components: location of the previous vehicle, maximum radio range, zone type to which the vehicle is associated (constructed based on the angular information), and distance between vehicles with respect to the entity that generated the event. Upon reception of the DCN message, the receiving vehicle computes the zone in which it lies, considering the previous vehicle location from which it received the message.

**Figure 3:**
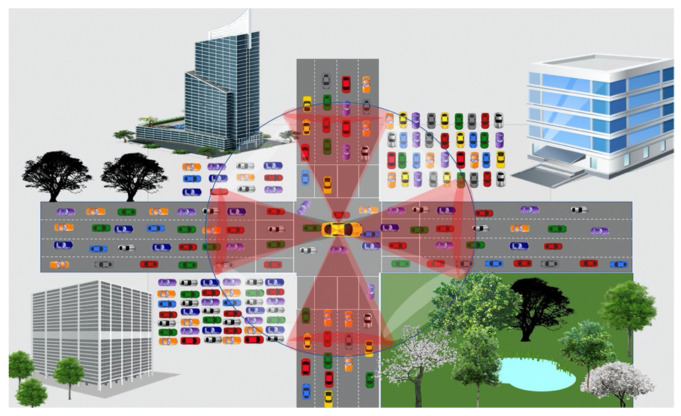
Communication range divided into eight zones: 04 Red, and 04 White zones.

In our scheme, there are two types of zones: red and white. As shown in Figure 3, each receiving vehicle divides the area of communication (1-hop) into eight zones, each of 45 degrees. The four zones with the following ranges are termed as red zones: (i)>=67.5 and≤112.5, (ii) >=157.5 and≤202.5, (iii) >=247.5 and≤292.5, and (iv) ≤22.5 and>=337.5. Red zones are the most significant zones due to the following reasons: (i) in these zones, most vehicles are mobile, (ii) being the middle zones (in the vehicle’s radio range), selecting forwarders first from these zones will help reduce unnecessary retransmission. The remaining four zones are termed white zones. The suppression angle concept is introduced to provide priority to vehicles located in better positions on roads. Exploiting the previous vehicle location from which the current vehicle received the message, the vehicle computes the angle information (Equation (5)).

(5) Angle_PrevNode←AngleCompute(DCN_msg.PrevNodeLocation, CurrentLocation())

Using the angle information, the vehicle figures out whether it lies in the red zone or the white zone. To address the broadcast storm problem due to the broadcast of DCN messages, forwarding priorities are computed by all vehicles that lie inside the transmission region based on information including zone type, the distance between vehicles with respect to event vehicle (that generated the event), and maximum radio range ($RR_{max}$). For such purpose deferred timer concept is exploited.

When a vehicle broadcasts the DCN message, all vehicles in the vicinity receive the message. If it is not a duplicate message, meets spatial and time validity, every vehicle sets a deferral timer. Red zones vehicles are given priority over white zone vehicles. A deferral timer is set based on geographic progress toward the destination and the type of zone in which it lies. This allows the eligible receiving vehicles to contend for the forwarder. Shorter delays are calculated by the vehicles in red zones compared to vehicles in white zones, as shown in Algorithm 1. A small delay is added to the computed deferred timer value of the vehicles in white zones. The aim is if they overhear the duplicate message from vehicles, then they should suppress it; otherwise, they send it upon timer expiry.

The geographic progress toward the destination (where spatial validity expires) is computed based on two information components: (i) distance of the previous vehicle from the hazardous site location (Equation (6)), and (ii) distance of the current vehicle from the hazardous site location (Equation (4)). The farthest vehicle(s) that lies in the red zone(s) are given priority by computing shorter delays (waiting time) for them (Equation (7)). During the waiting time, the vehicle listens to the channel. If it overhears the DCN message with the same content name, then it stops the timer.

(6) $$PrevNode_{DistHazard}\leftarrow Distance\;\left(\begin{array}{c} DCN\_msg.CACN.Where.Location\;,\;\\ \;DCN\_msg.PrevNodeLocation \end{array}\right)$$

(7) $$FrwdTime\leftarrow Delay_{Max}*\left(1-\;\frac{\;{PrevNode}_{DistHazard}-\;{CurrNode}_{DistHazard}}{RR_{max}}\right)+\;Delay_{random}\left(0,i\right)$$

## 6. Performance Evaluation

For evaluation of our proposed scheme called DCN protocol, which exploits the CACN naming scheme, we implemented it in ndnSIM [40,41]. To create mobility scenarios, we distributed a set of (50 primarily) vehicles randomly on the road of 1.8 km. Different events on the road (e.g., road work, post-crash notification, etc.) can significantly affect traffic conditions. Dissemination of event notification to vehicles heading to the event site may help to make timely decisions such as to reroute or reduce speed. In most safety applications, only a message containing CACN will be sufficient for vehicles to make a timely decision. For example, in safety applications such as dangerous road warning, work zone warning, emergency vehicle warning, and post-crash notification, all vehicles moving to the hazardous site need a contextual alert. Such an alert will be adequate for most of the vehicles, reducing the need to send explicit interest messages to request data. We compare our scheme performances with the most recent proposals: hierarchical name-based (HNB) [23] and extended content-centric network (ECCN) [39].

**Figure 4:**
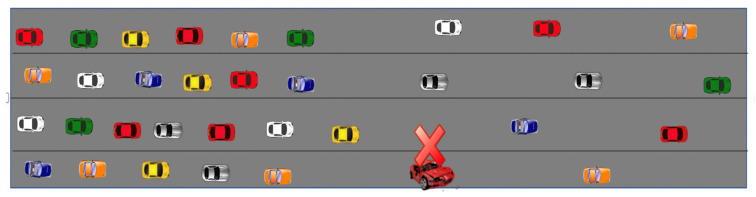
Simulation scenario.

Our proposed DCN scheme considers a push-based scheme for the dissemination of notification messages containing context-aware content names. The schemes presented in the works of [23,39] exploit a push-based approach for the dissemination of safety content. Therefore, we selected these two closely relevant schemes for comparison. Another reason for selecting the work of [23] is that it also proposes a naming scheme for vehicular communication. We considered the safety application scenario as considered in the work of [23,39]). Figure 4 presents an experimental scenario where an accident vehicle broadcasts the message to the entities (RSU, vehicle) in its neighborhood. In our proposed DCN protocol, a message is comprised of two fields: CACN and the location of the previous forwarder vehicle. The size of the DCN message is 83 bytes. Whereas in the case of HNBs [23] and ECCN [39], the message is a data message with content of size 1103 bytes. Just like the work of [23,39], we assume that each vehicle is equipped with GPS, using which it obtains its location. In the simulation scenario, we use an 1800 m road, with one accidental vehicle as a producer. Common parameter settings for all the experiments are depicted in Table 2.

In the work of [39], hop count is used to control the dissemination of the data message, whereas, in the work of [23], the area-of-interest (RSU radio range) concept is exploited. In addition, no suppression scheme is proposed to address the broadcast storm problem. In the work of [23], no suppression scheme is incorporated to address the broadcast storm problem. In the work of [23], whenever a vehicle receives a data packet, it will participate in its dissemination if it is lying in the RSU radio range whose ID is mentioned in the content name. Each time upon receiving the same (duplicate) message from the neighbors, a vehicle reforwards it as long as it satisfies the validity check (lying in the RSU range whose ID is mentioned in the content name). Depending on the vehicle speed and radio range of the RSU, a vehicle resends the same (duplicate) message a large number of times. In the work of [39], hop count is used to control the dissemination of the data message. In addition, no suppression scheme is proposed to address the broadcast storm problem. In the work of [39], when a vehicle receives a data packet, it will participate in its dissemination only if the TTL in the packet is greater than zero. Each time upon receiving the same (duplicate) message from the neighbors, a vehicle reforwards it after decrementing the TTL. Each time when a vehicle receives the same data message from one or more of its neighbors (with identical or different TTL), it will reforward it (if it meets the validity check) after decrementing the TTL. In our work, each vehicle can participate in the dissemination of the received DCN message only if it meets the spatial and time validity. Furthermore, our scheme incorporates a suppression scheme to address the broadcast storm problem. In all the experiments for our proposed DCN protocol, we considered the spatial validity as 900 m and time validity as 10 s. For ECCN [39], we set the time to live (TTL) requirement to 15. Each experiment scenario evaluation is repeated 10 times, and the results are plotted after taking the average.

### 6.1. Experiment 1: Total Number of Messages Processed vs. Number of Packets Created Per Second

**Figure 5:**
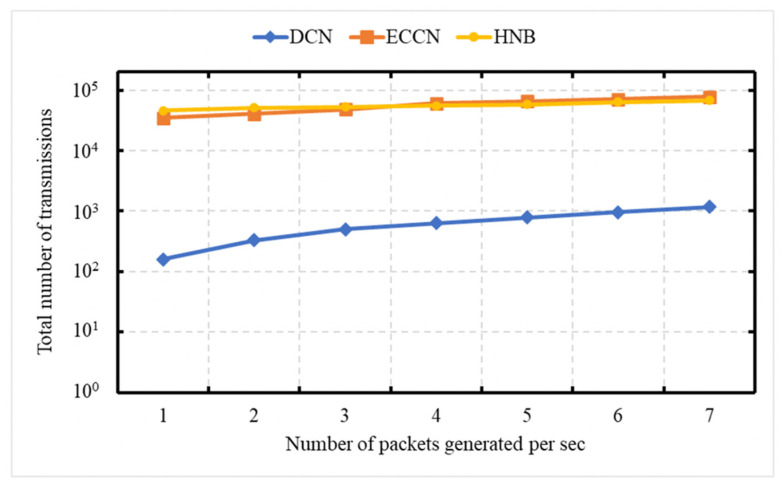
Total number of messages generated per second.

The experiment is carried out to demonstrate the network load in terms of the total number of transmissions (data [23,39] or DCN messages) as a function of the number of messages generated by the event vehicle per second. This experiment is conducted over a network of 50 vehicles, with an average speed of 72 kph. In this experiment, the number of messages generated by the event vehicle ranges between 1 and 7 packets/s. The X-axis is representing the number of messages generated by the event vehicle per second, and the Y-axis represents the number of transmissions. The Y-axis uses a logarithmic scale [49]. Figure 5 shows the comparison of our DCN protocol with HNB [23] and ECCN [39]. The schemes presented in the works of [23,39] exploit a push-based mechanism for the dissemination of safety content. In HNB [23], only those vehicles continue participation in dissemination that lies in the radio range of RSU, whose ID is mentioned in the content name. No message suppression scheme is presented. If a vehicle receives the data packet and is not in the radio range of a specific RSU, it discards it. In ECCN [39], each vehicle participates in the dissemination of safety content if the TTL requirement is satisfied. In contrast, in our proposed DCN protocol, the event vehicle only disseminates the notification message containing context-aware content name. Each vehicle participates in its dissemination as long as it satisfies the spatial validity and time validity. In addition, a suppression scheme is incorporated to address broadcast storm problems (by controlling the dissemination of duplicate messages). In Figure 5, it is obvious that with an increase in the number of messages generated by the event vehicle per second, there is an increase in the number of messages generated for DCN, HNB [23], and ECCN [39]. The proposed scheme (DCN) minimizes the number of messages disseminated in the network and can be considered as a solution for the dissemination of notification information related to safety content.

### 6.2. Experiment 2: Total Number of Messages Processed with Relative Speed

**Figure 6:**
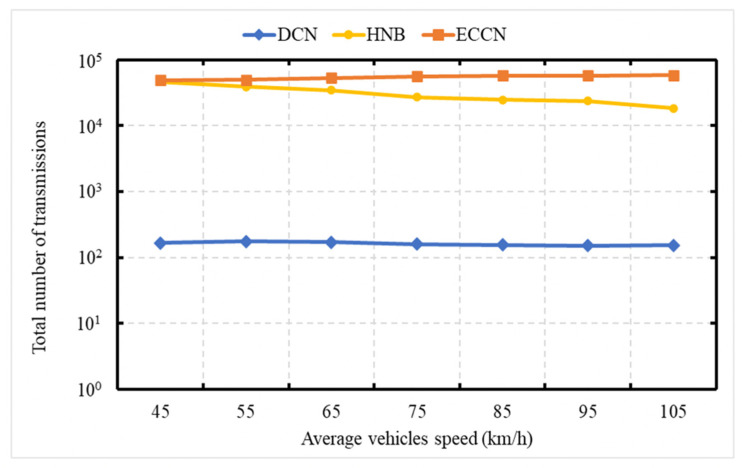
Total number of messages processed with relative speed.

The experiment is carried out to demonstrate the network load in terms of the total number of transmissions as a function of the vehicle’s speed. This experiment is conducted over a network of 50 vehicles with varying speeds ranging from 40 to 120 kph. The X-axis is representing the average vehicle speed, and the Y-axis represents the number of transmissions. In Figure 6, the vertical axis (Y-axis) uses a logarithmic scale [49]. Figure 6 shows the comparison of our DCN protocol with HNB [23] and ECCN [39]. In this experiment, the event vehicle generates one message per second. In the case of HNB [23], due to an increase in vehicle speed, it stays in the RR of the RSU for a short duration. In the work of [23], the area-of-interest (RSU radio range) concept is exploited to control the dissemination of the data message. The vehicle participates in the dissemination only if it lies in the area of interest. Therefore, with an increase in vehicle speed, there is a decrease in the number of messages for HNB [23]. For DCN, an increase in speed does not impact the number of message transmissions, and the reason is that a node participates a limited number of times due to the proposed suppression scheme. In the work of [39], hop count is used to control the dissemination of the data message. Due to increased speed, a dynamic environment is generated that restricts interconnectivity between vehicles for a longer duration. Consequently, there is a slight increase in the number of transmissions with the speed increase.

### 6.3. Experiment 3: Total Number of Messages Processed vs. Number of Vehicles

**Figure 7:**
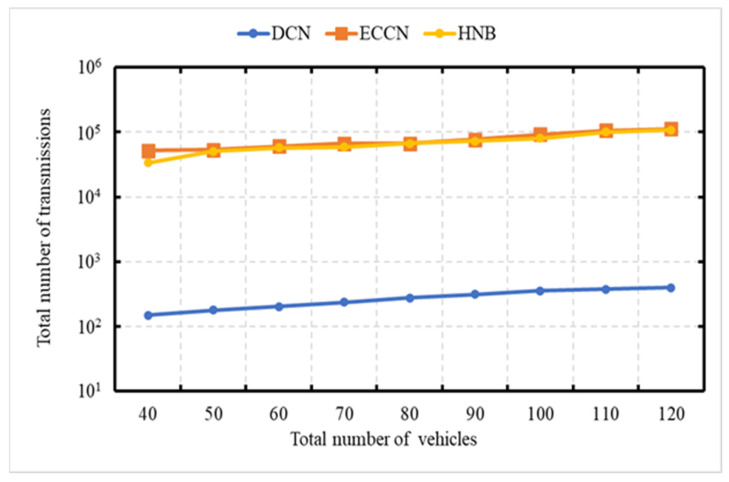
Total number of messages processed as a function of the number of vehicles.

The experiment is carried out to demonstrate the network load in terms of the total number of transmissions as a function of the number of vehicles. This experiment is carried out over the networks with 40, 50, 60, 70, 80, 90, 100, and 120 vehicles, with an average speed of 70 kph. The X-axis represents the number of vehicles in the network, and the Y-axis represents the number of transmissions. In Figure 7, the vertical axis (Y-axis) uses a logarithmic scale [49]. In this experiment, the event vehicle generates one message per second. Figure 7 shows a performance comparison of our DCN protocol with HNB [23] and ECCN [39]. It is obvious to see that with an increase in the number of vehicles, there is an increase in the number of messages for DCN, HNB [23], and ECCN [39]. In the proposed DCN, the message dissemination is controlled; therefore, the trend is not as steep as that of ECCN [39] and HNB [23].

### 6.4. Experiment 4: Total Energy Consumption

**Figure 8:**
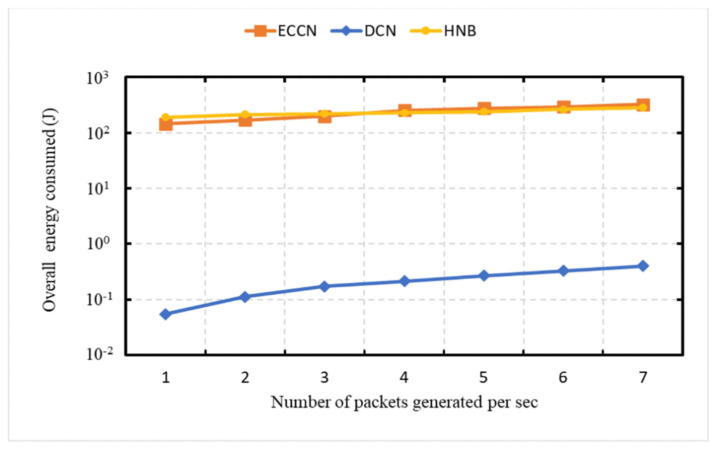
Total energy consumption by the increasing number of messages generated per second.

**Figure 9:**
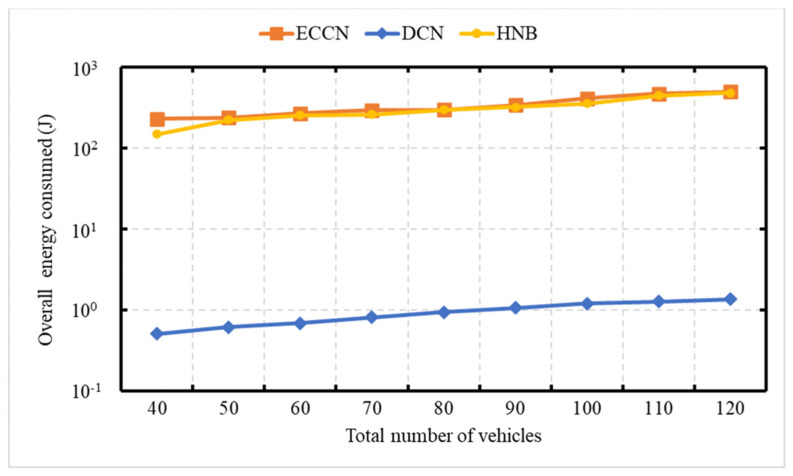
Total energy consumption increasing network size.

**Figure 10:**
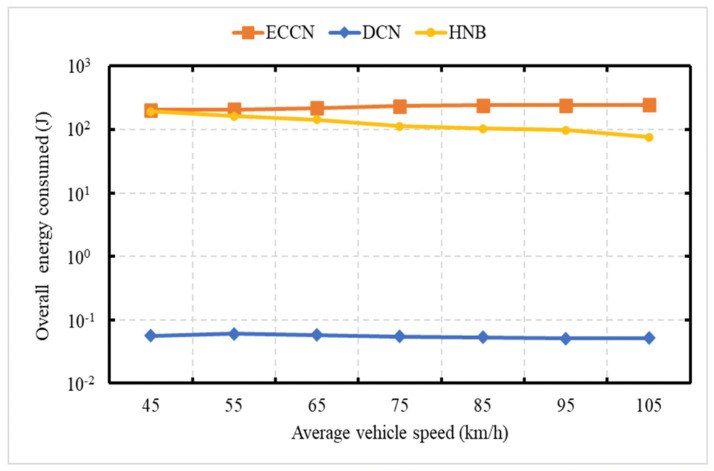
Total energy consumption considering the average relative speed of vehicles.

In this experiment, we demonstrate the scalability property of our proposed DCN protocol. We conducted a set of experiments (Figure 8, Figure 9 and Figure 10) to demonstrate the overall energy consumption in the network. In Figure 8, Figure 9 and Figure 10, the vertical axis (Y-axis) uses a logarithmic scale [49]. For this experiment, we consider the energy model presented in the work of [23,50] for energy consumption evaluation. Figure 8 demonstrates the energy consumption as a function of the number of messages generated by the event vehicle per second. In Figure 8 and Figure 10, the number of vehicles considered is 50. In contrast, 75 kph is considered as average vehicle speed. In Figure 8, it can be seen that with an increase in the number of messages generated by the event vehicle per second, there is an increase in the energy consumption for DCN, HNB [23], and ECCN [39].

Figure 9 demonstrates the energy consumption as a function of the network size. The average speed of vehicles is 75 kph. In Figure 9 and Figure 10, the event vehicle generated one message per second. With an increase in the number of vehicles, there is an increase in the number of messages, resulting in an increase in energy consumption for DCN, HNB [23], and ECCN [39]. Figure 10 demonstrates the overall network energy consumption as a function of an increase in the average vehicle speed. In this experiment, the event vehicle generates one message per second. It is obvious to see that, with an increase in vehicle speed, there is a decrease in the energy consumption for DCN, HNB [23], and ECCN [39]. In contrast, the energy in the case of ECCN presents a slightly increasing trend.

### 6.5. Experiment 5: Network Load

**Figure 11:**
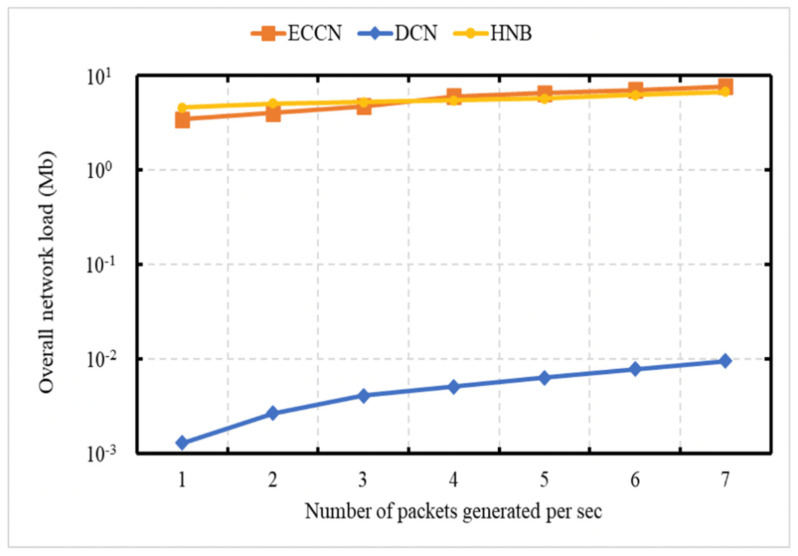
Total network load as a function of the number of messages.

**Figure 12:**
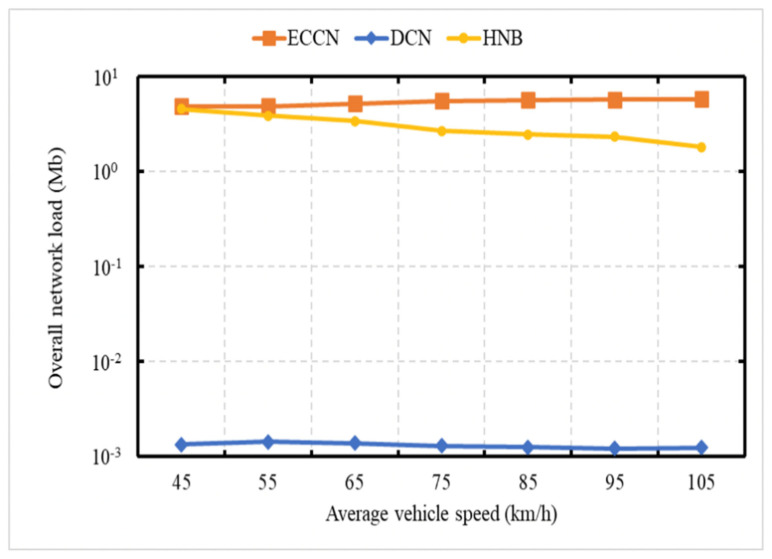
Total network load as a function of average vehicle speed.

**Figure 13:**
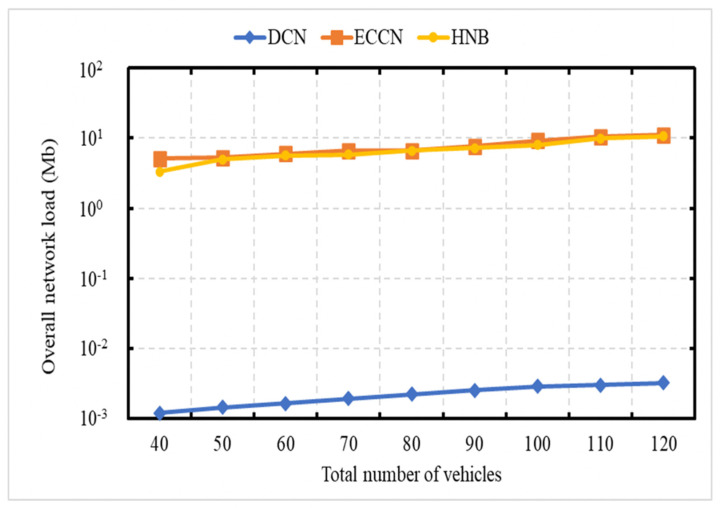
Total network load as a function of the number of vehicles.

In this experiment, we demonstrate the scalability property of our proposed DCN protocol. We conducted a set of experiments (Figure 11, Figure 12 and Figure 13) to demonstrate the network load. In Figure 11, Figure 12 and Figure 13, the vertical axis (Y-axis) uses a logarithmic scale [49]. Figure 11 demonstrates the network load as a function of the number of messages generated by the event vehicle per second. This experiment is conducted over a network of 50 vehicles, with an average speed of 75 kph. In this experiment, the number of messages generated by the event vehicle ranges between 1 and 7 packets/s. It is obvious to see that, with an increase in the number of messages generated by the event vehicle per second, there is an increase in the network load for DCN, HNB [23], and ECCN [39].

Figure 12 demonstrates the network load as a function of the average vehicle speed. This experiment is conducted over a network of 50 vehicles with varying speeds between 40 and 120 kph. In Figure 12 and Figure 13, the event vehicle generates one message per second. It is obvious to see that, with an increase in vehicle speed, there is a decrease in the network load for HNB [23]. Figure 13 demonstrates the network load as a function of the network size. It is obvious to see that, with an increase in the number of vehicles, there is an increase in the network load for DCN, HNB [23], and ECCN [39].

### 6.6. Experiment 6: Delay

**Figure 14:**
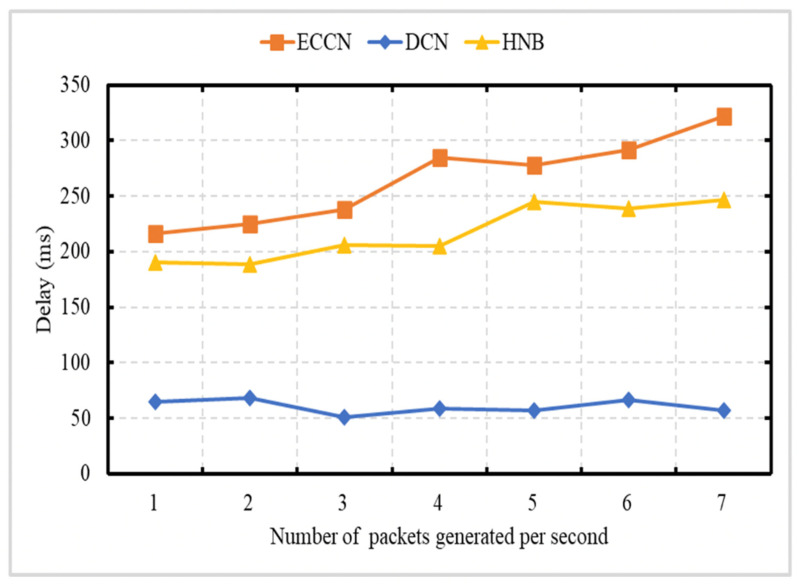
Average delay as a function of the total number of messages generated per second.

Figure 14 illustrates the average delay of DCN, HNB [23], and ECCN [39] schemes in a VANETs scenario. This experiment is conducted over a network of 50 vehicles, with an average speed of 75 kph. In this experiment, the number of messages generated by the event vehicle ranges between 1 and 7 packets/s.

Figure 14 shows that HNB [23] and ECCN [39] face higher delays compared to our proposed DCN scheme. This is due to an increase in congestion due to a higher number of packets in the network. DCN protocol is based on CACN and exploits the receiver-based suppression scheme. The DCN protocol allows the packets to reach the vehicles coming toward the event site with minimum delay.

## 7. Conclusions

Different events on the road (e.g., road work, post-crash notification, etc.) could have a big impact on traffic conditions. Dissemination of information about such events to all vehicles heading toward the event site may help them decide whether rerouting or reducing speed is necessary. It is required to disseminate the information to all the vehicles heading toward the event site within a short time. To disseminate safety content information, there exist literature that exploits push-based data dissemination technique in NDN. Such schemes though meet the low latency requirement but result in a broadcast storm problem in the dense NDN-based VANETs. In most safety applications, only a contextual alert containing a context-aware name will be enough for vehicles to make the decision timely. In NDN, content name plays a vital role in effective and efficient content forwarding and caching.

In this paper, the context-aware content-naming (CACN) scheme is presented to provide an adaptive content dissemination solution in NDN-based VANETs. The focus is making dynamic decisions related to forwarding, cache management, etc. (based on content context, spatial validity, time validity information) rather than treating each content in the same way. In the content name, the context information is aggregated along with the information components such as: type of content (such as safety and non-safety), scope, application type (e.g., post-crash notification), content format (text, audio, and video), the location where content is generated, the time when content is generated, and network environment (such as road type (rural, urban, highway) where the vehicle is located). CACN allows naming the safety and non-safety contents. The CACN scheme aggregates the context and content-specific information. In addition, a coding scheme is presented to represent most of the content name components, which enables addressing communication and storage complexity. 

To overcome the broadcast storm problem, we presented a receiver-based DCN protocol that exploits angle information, distance, zone, spatial validity, and time validity information to select a forwarder. We have demonstrated by simulation that the proposed DCN protocol reduces unnecessary DCN message retransmissions. Furthermore, due to the context-aware content name (CACN) in the message, the vehicle can make decisions more quickly, as it reduces the need in the majority of the safety applications to access the data packet explicitly. Very few NDN-based VANETs naming schemes exist, but these cannot cater to a wide range of VANETs applications. In the future, there is a need for NDN-based VANETs naming schemes that focus on the context-aware naming of both safety and non-safety applications.

## Figures and Tables

**Table 1:** A precise description of existing proposed naming strategies.

Format	Ref.	Strengths
/traffic/geo-location/timestamp/type of data/nonce/	[15]	Use the traffic type component at the start of the name prefix Included spatial and temporal scope of traffic information Datatype component represents the type of data, e.g., speed, closed lane, etc.
/NDN/bit/parking/……./$c_{1}/c_{2}/../c_{n}$	[16]	Data naming structure for traffic information dissemination Uni-dimensional naming scheme to map bi-dimensional geographic areas
/Dest-L(1)…--: Dest- L(n) : end/type : … : type : end/ Source-L=type&data : … : type&data : end/next : … : next :End/	[17]	Three-level hierarchical geolocation-based method Scale-based aggregation
/high/trafficInfo/streetXy/km20/	[18,19]	Presented forwarding schemes that forward based on the content priority Vehicular data traffic is divided into two priority categories, i.e., high and low
/application/geo_reference/temporal_field/nonce/	[20]	Geo-tagged name-based information retrieval Opportunistic forwarding strategy
/application_prefix/datatype/data_location/name_marker/vehicle_name/timestamp/	[21]	Scheme to identify false information dissemination malicious vehicle
/top_level_category/class_category/application/primary_identifier/contextual_identifier/additional_tags/	[22]	Naming solution based on application classification. Applications are divided into three categories: (i) safety, (ii) transit, and (iii) infotainment Support quality of service management. The application component identifies the type of services Parameters that can impact communication are considered, such as speed and direction
/Hongik/BuildingA/Floor3/ Room301/Id001/Vehicle-ID/RSU-ID/direction/speed/	[23]	Application and content-related information not considered Parameters that can impact communication are considered, such as speed and direction Scheme to control the broadcast storm
hmn://traffic/highway/safety/video: 629d5f3f1f0a112f: /time/bitrate/ public/emergency/	[24]	The name includes two hierarchical components and one flat component. The flattish component uniquely identifies the content Contextual information about the content includes time, priority level, and caching strategy Naming scheme designed for naming multimedia contents
vhn://us/nv/lv/smith/NVD0001/text/:::=uSwjkHZ	[25,26]	The name includes two hierarchical components and one flat component Name includes information about the producer but not about the application
/longitude/latitude/application/timestamp	[27]	To efficiently forward interest packets To control the broadcast storm To retrieve the data from the area of interest generated at a specific time
/category/service_name/additional_info/	[28]	Bloom filter-based content discovery Naming based on the content popularity and shareable level Data services are classified into three categories: popular public, popular private, and unpopular

**Table 2:** Common configuration parameters.

Parameter	Value
No. of RSUs	1
Producer Vehicle	1
Vehicle Radio Range (RR)	250 m
RSU RR [23]	200 m
Road Length	1800 m
Number of hops [39]	15
Event node placement	900 m
Propagation Loss Model	Nakagami propagation loss model
Technology	IEEE 802.11a
Energy Consumption	0.5 µJ/bit
Caching Policy	LCE
Replacement Policy	LRU
Simulation Time (s)	1200 s
TxPowerStart	5 (dbm)
TxPowerEnd	5 (dbm)
Packet Length	1103 bytes for HNB [23], ECCN [39] with content.83 bytes for DCN [proposed scheme]
Vehicle speed	75 kph (unless specified explicitly)
Number of vehicles	50 (unless specified explicitly)
